# It Takes a Village: Sources of Spiritual Care in Hospice That Support the Well-being of African-American Hospice Patients and Their Caregivers in the USA

**DOI:** 10.1007/s10943-025-02548-4

**Published:** 2026-01-18

**Authors:** Denise D. Quigley, Sara G. McCleskey, Jason Lesandrini, Natalie McNeal, Nabeel Qureshi

**Affiliations:** 1 RAND Corporation, 1776 Main Street, Santa Monica, CA 90401 USA; 2 Wellstar Health System, Marietta, GA USA; 3 Hospice of Northeast Georgia Medical Center, Gainesville, GA USA

**Keywords:** Spiritual care, Well-being, Quality of life, Hospice care, Clergy, Chaplains, Bereaved caregivers

## Abstract

Spiritual care offers significant benefits to hospice patients and caregivers. However, disparities exist in the perceived quality of spiritual care among African-American caregivers. We interviewed bereaved caregivers of African-American decedents, their chaplains and clergy, and conducted a medical record review to explore the sources of spiritual care that support well-being for African-American patients and caregivers in hospice. We found that both chaplains and clergy supported social and spiritual well-being, whereas other hospice team members were seen as responsible for the hospice environment and physical well-being. Church congregations and families were most involved in providing social and emotional support.

## Introduction

**Fig. 1:**
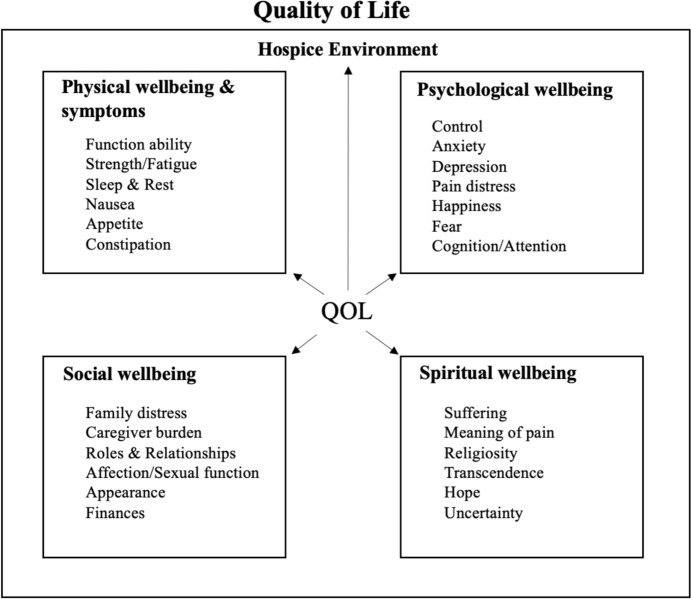
City of Hope Quality of Life (QOL) Model, Adapted*. NOTE: QOL indicates quality of life. * indicated that we adapted the original model. We adapted the model only by adding the words “hospice environment” and adding an arrow from QOL to Hospice Environment. We did this to indicate that QOL is for those in a hospice (rather than another setting of care). Source is Ferrell, B. R. (2017). Spiritual care in hospice and palliative care. *The Korean Journal of Hospice and Palliative Care*, 20(4), 215–220

Spiritual well-being is recognized as an important dimension of overall quality of life (QOL) in hospice care, which according to the City of Hope QOL model, consists of four domains of well-being: physical well-being and symptoms (Jim et al., 2015); emotional well-being (Bultz et al., 2011; Whitford et al., 2008); social well-being (Sherman et al., 2015; Sun et al., 2016); and spiritual well-being (Ferrell, 2017; Ferrell et al., 2015). As shown in Fig. 1, these QOL domains are interrelated and work together to influence all aspects of the patient and caregiver (that is, primary, informal caregiver) hospice care experiences.

Spiritual care, or the professional attention paid to spiritual, religious, or existential needs of patients and caregivers, is crucial in hospice (Ramezani et al., 2014). Evidence suggests that spiritual care has multiple benefits for patients and caregivers (Daaleman & VandeCreek, 2000; Flannelly et al., 2003; VandeCreek & Lyon, 1995), as it may help patients cope with their illness, alleviate symptoms of distress, and improve emotional/social outcomes (McClement et al., 2007; Pargament, 1997; Rippentrop et al., 2005), and help caregivers by reducing distress and mediating grief (Anderson et al., 2008; Kiely et al., 2008).

National clinical practice guidelines for hospice in the USA describe the general provision of spiritual care as involving an interdisciplinary team who, either directly, through referral, or with a professional chaplain, “facilitates spiritual and cultural rituals or practices as desired by the patient and family” (National Coalition for Hospice & Palliative Care, 2018). This interdisciplinary team can include physicians, nurses, social workers, support staff, and hospice chaplains. Hospice patients and their caregivers may also have existing relationships with clergy or church community members who provide spiritual support at the end of life and while on hospice.

While spiritual care is widely recognized as vital to the provision of person-centered care, especially at the end of life, the perceived quality of spiritual care in hospice varies by several factors. In particular, it varies by race. Caregivers of African-American hospice patients more often express dissatisfaction with the spiritual care provided in hospice compared to caregivers of White hospice patients (Rhodes et al., 2012). This disparity may be due to several factors, including lack of racial concordance between patients and clinicians, patient or caregiver experiences with discrimination, lack of culturally relevant spiritual care by hospice chaplains, or limited familiarity with or training in hospice care among religious leaders (Crawley et al., 2000; Payne, 2001). Religious and spiritual needs may also differ by race, with evidence suggesting that African-American patients utilize religious coping to a greater degree than White patients and may report more significant spiritual needs (Astrow et al., 2018; Johnson et al., 2005). Also, African-American patients may rely more heavily on spiritual support outside of the hospice team, through religious leaders and church communities, when compared with White patients (Sloan et al., 2021). However, the nature of these factors and the role of community clergy within the wider care team remains less clear.

Despite the importance of spiritual care in hospice and evidence of racial disparities in experiences with spiritual care delivery, there has been little examination of spiritual care delivery in hospice specifically for African-Americans. Guided by the City of Hope QOL model (Bultz et al., 2011; Ferrell, 2017; Ferrell et al., 2015; Jim et al., 2015; Sun et al., 2016; Whitford et al., 2008) (Fig. 1), we explored African-American experiences of spiritual care in hospice by describing how and by whom spiritual care is delivered to hospice patients and their caregivers, and by examining how spiritual care provision supports overall QOL in hospice.

## Methods

### Setting

This study was conducted in a large, urban/suburban community hospice within a health system located predominantly in and surrounding Atlanta, Georgia. In 2024, Atlanta, Georgia was a majority–minority city (47.9% Black or African-American compared to 39.9% White) (US Census Bureau, Undated) and most individuals (64%) identified as Christian, a plurality of whom (42%) identified as Evangelical Protestant (Pew Research Center, 2025). The hospice provides care at home and in two 18-bed hospital inpatient units to approximately 1700 patients annually and employs two full-time physicians (board certified in palliative care), 80 registered nurses, four licensed practical nurses, nine social workers, and five chaplains. They monitor care quality using the Consumer Assessment of Healthcare Providers and Systems Hospice survey.

### Sample

Detailed sampling information has been published elsewhere (Quigley et al., 2024). Briefly, in 2023, the hospice identified bereaved caregivers of African-American hospice decedents who died from October to December 2022 (*n* = 58). During routine 90-day bereavement support calls, hospice bereavement coordinators obtained study interest, consent to pass on contact information, and asked whether the caregiver would allow the hospice to share the decedent’s medical records with the study team for review. Forty percent (23/58) of the sample agreed to provide contact information and decedent records to the study team. We recruited decedent/caregiver dyads balancing on place of death (home, inpatient unit), length of hospice stay (less than 5 days, 5–12 days, 13 or more days), spiritual background of the decedent (none and various Christian denominations), and relationship of decedent/caregiver (decedent was parent, spouse, friend) and completed interviews with 12 caregivers (12/23; 52% response rate).

The hospice administrator emailed hospice chaplains with study information and a request to provide their contact information to the study team. All five chaplains agreed and completed interviews. Community clergy were identified by caregivers during interviews (that is, the interviewer asked whether the caregiver was willing to share clergy contact information). Only clergy physically present at the hospice bedside were recruited (3 of 5 named clergy); all three clergy completed interviews.

### Data Collection

We conducted 20 one-hour semi-structured phone interviews that included hospice chaplains employed at the hospice (February/March 2023; *n* = 5); community clergy who ministered directly to bereaved caregivers and/or decedents (that is, physically present at the hospice) during their hospice stay (June/July 2023; *n* = 3), and bereaved caregivers (May/June 2023; *n* = 12). The lead author conducted all interviews with a second member of the study team (SM or NQ) taking notes.

Chaplains were asked about their experiences delivering spiritual care at the hospice, their experiences providing care to diverse communities in general, and their interactions with community clergy. Clergy were asked about experiences working in hospice and their interactions with bereaved caregivers and decedents. Bereaved caregivers were asked about their perceptions of hospice; the practical, emotional, and spiritual support they received; and any challenges they encountered with hospice care. Similar questions were asked across interviews to understand differences in spiritual care from the perspectives of spiritual care providers (chaplains and clergy) and beneficiaries (caregivers). Interview protocols are available upon request; interviews were recorded and transcribed verbatim.

Hospice administrators transmitted de-identified medical records (including notes and number of visits by care team members and clergy) using a Secure File Transfer Protocol. We reviewed decedent records (*n* = 23) and coded information about the emotional, religious, and spiritual support provided based on the narrative text notes that document care plan needs, care received, and by whom care was provided that were entered by each care team member.

### Analytic Approach

We entered transcripts and medical records into Dedoose Version 9.2.22 (SocioCultural Research Consultants, 2018). We established initial codes mapped to key research questions (Bernard & Ryan, 2010) and extant literature on emotional, spiritual, and religious support in hospice and care disparities at the end of life (Daaleman et al., 2008; Edwards et al., 2010; Hvidt et al., 2020; LeBaron et al., 2016) (deductive process, Fig. 1). We further developed the code structure using systematic, inductive procedures to generate insights from responses (Bradley et al., 2007; Thomas, 2006) for each data source (that is, interviews with chaplains, clergy, and caregivers and decedent medical records).

Three members of the study team (DDQ, SM, and NQ) coded transcripts to identify relevant themes (Krippendorff, 2019), coding early transcripts independently and refining the codebook (Bernard & Ryan, 2010). We used regular team meetings to identify discrepancies, refine concepts, discuss concepts and themes, and reach consensus on topics (Miller & Crabtree, 1999). We compared differences in code application among coders across three transcripts, obtaining a pooled kappa coefficient of 0.89 for chaplain, 0.91 for clergy, and 0.86 for caregivers, indicating “very good” agreement (McHugh, 2012). Remaining transcripts were divided among the researchers and coded independently. We employed ongoing training among the coding team on emerging subcodes using the Dedoose training module.

We conducted thematic analysis across interviews to identify themes related to spiritual care provision and comparative analysis to understand differences in approach by different spiritual care providers. We identified types of spiritual care provided to decedents and caregivers, mapping the aspects of spiritual care to the individual who provided it based on caregiver, chaplain, and clergy-reported spiritual care in interviews and signed patient notes in the medical record. Spiritual care providers were categorized into three groups: hospice (hospice generally, chaplain, or registered nurse and social worker on care team); church (clergy or church family); and family (family and caregiver of the decedent or the decedent themselves).

Study protocols were approved by RAND’s Human Subjects Protection Committee (IRB Assurance No.: FWA00003425; IRB No.: IRB00000051; Project ID: 2023-N0018).

## Results

### Chaplain and Clergy Characteristics

We interviewed five staff chaplains and three community clergy. Chaplains and clergy were predominantly male and Baptist, with all chaplains identifying as White and all clergy identifying as African-American (Table 1). All decedents were African-American, on average 75 years old, spent approximately two weeks in hospice, and identified themselves as primarily non-denominational Christian (that is, Christian not affiliated with a traditional denomination) or Baptist (Table 2). Most of the caregivers were adult children or partners of the decedent.

**Table 1:** Participant demographic characteristics of hospice chaplains (*n* = 5) and community clergy (*N* = 3)

Characteristic	Hospice Chaplain (*n* = 5)	Community Clergy (*n* = 3)
	Mean (range)	Mean (range)
Age	58.8 years (42–70)	60.3 years (42–75)
Years in hospice care	15.5 years (7.5–26)	NA
Years at named hospice	7.1 years (3–11)	NA
Years as a pastor	NA	22.3 years (17–30)

	% (*N*)	% (*N*)
Male	80% (4)	100% (3)
Race		
Percent White	100% (5)	0% (0)
Percent African-American	0% (0)	100% (3)
Religious background		
Non-denominational Christian	20% (1)	33% (1)
Baptist	60% (3)	67% (2)
Methodist	20% (1)	0% (0)
Worked at another hospice	80% (4)	NA

**Table 2:** Participant Characteristics of African-American Decedents and their Caregivers (N = 12), Overall and By Caregiver Reported Adequate or Inadequate Spiritual Care

Characteristic	Data source	African-American Decedents (*n* = 12)	African-American Decedents Whose Caregivers Reported *Adequate* Spiritual Care in Hospice (*n* = 7)	African-American Decedents Whose Caregivers Reported *Inadequate* Spiritual Care in Hospice (*n* = 5)
		Mean (SD)	Mean (SD)	Mean (SD)
Decedent Age in years	EHR	74.8 (14.3)	78.0 (5.5)	69.7 (8.8)
Time in hospice in days	EHR	12.9 (7.3)	13.2 (7.4)	11.8 (0.1)
Time in hospice in days	Interview	13.8 (10.4)	13.7 (7.9)	13.8 (6.6)

		% (*N*)	% (*n*)	% (*n*)
Decedent Religion	Interview			
Non-denominational Christian		42% (5)	14% (1)	80% (4)
Baptist		33% (4)	57% (4)	0% (0)
Methodist		8% (1)	0% (0)	20% (1)
Jehovah’s Witness		17% (2)	29% (2)	0% (0)
Decedent Discharge Disposition	EHR			
Expired in inpatient hospice unit		67% (8)	71% (5)	60% (3)
Expired at home		33% (4)	29% (2)	40% (2)
Relationship to Caregiver	EHR			
Parent		50% (6)	57% (4)	40% (2)
Spouse/Fiancé		33% (4)	29% (2)	40% (2)
Sibling		8% (1)	0% (0)	20% (1)
Friend		8% (1)	14% (1)	0% (0)
Chaplain Care	Interview			
Offered Chaplain Care		75% (9)	86% (6)	60% (3)
Wanted Chaplain Care		92% (11)	86% (6)	100% (5)
Got Chaplain Care		67% (8)	71% (5)	60% (3)

EHR indicates electronic health record. Adequate indicates it was the “right amount”; inadequate indicates it was “too little”

### Visits

On average, hospice patients were visited by clergy once and a social worker once, with two visits from their physician/nurse practitioner. Nurses rounded on inpatient unit hospice patients every two hours and those patients at home on average had 7 nursing visits and 4 visits by aides. We found similar visit patterns for those who completed interviews and those who did not (data not shown).

### Provision of Spiritual Care that Supports Well-being

We identified four themes related to the sources of spiritual care that support well-being for African-American decedents and caregivers in hospice. Themes 1 and 2 describe how and by whom spiritual care is delivered. Themes 3 and 4 examine how spiritual care in hospice supports the spiritual, emotional, social, and physical well-being of African-American hospice patients and caregivers.

*Theme 1*: Chaplains and clergy both reported engaging regularly in active listening, making connections, and building rapport. Chaplains, however, more frequently described asking open-ended questions, emphasizing sensitivity to cultural needs, and focusing on being present. Clergy more often reported engaging in strategies to facilitate reconciliation and conducting life review.

Chaplains and clergy engaged in a variety of strategies to deliver spiritual care. Both highlighted the importance of active listening. A chaplain explained, “*I listen for personal aspects, what community needs they have, what social aspects they point to and how they describe their relationship with other, with a higher power, with God*.” (Chaplain#4) Clergy also echoed this sentiment. One said, “*The number one skill is listening. There is a reason God gave us one mouth and two ears. That is a skill I am working on, but I am better at listening more. Sometimes you don’t have to say much, you just have to listen*.” (Clergy#003).

Making connections and building rapport were also important strategies used by chaplains and clergy. A chaplain said, “*I don’t use a checklist. I also don’t use a computer; that is a barrier. I ask them to tell me about themselves, what work they did, their life story. […] I think it is not good when you go in with a checklist – it is so cold. Spiritual care is conversational*.” (Chaplain#1) Another chaplain explained, “*Chaplains deal with talking about a person’s life, how they learned to cope. We try to use those established mechanisms that they point out in their life to get them through this time in hospice. We allow them to dictate to us a spiritual intervention*.” (Chaplain#3) Clergy highlighted the advantages of having an existing rapport with hospice patients from their time together prior to hospice. A clergy indicated:

*If I know them, I can go from conversations we had in the past, their favorite scripture, music, and be a comfort by being there. If I don’t know them, then I am a good listener, asking what they believe, what they know about the Bible, and go from there. If they don’t know much about the Bible, I start from their family, how they grew up. I try to get them at peace with not living much longer. -Clergy#002*

As per Table 4, while the type of spiritual care provided by chaplains and clergy was generally similar between the two, we also heard some differences. Chaplains more frequently reported asking open-ended questions, focusing on cultural sensitivity, and trying to be present. Chaplains pointed out that their approach to spiritual care is “dictated by the patient needs,” as chaplains are trained specifically to address the religious, spiritual, and cultural needs stemming from the range of patient and family beliefs (National Hospice and Palliative Care Organization, 2022b). A chaplain said, “*I am a blank slate. I don’t have an agenda, just questions*.” (Chaplain#3) Another chaplain described working with those of different religious backgrounds:

*Hispanic patients, the majority who are Catholic, tend to want sacraments, rosary. I try to have a Spanish-speaking chaplain who will come and pray with them and pray the rosary and whatever they need for sacraments. With African Americans and Hispanics, I try to think more about their needs based on their religious roots. We have some Hindu patients, some Jewish patients.[…] We try to connect people to their congregational religious needs with a faith leader of their own. But that adds to what is provided by hospice. Our spiritual care is dictated by the patient needs. -Chaplain#1*

Clergy, however, more often reported engaging in strategies to facilitate reconciliation on behalf of the patient and conducting life review. A clergy shared, “*My first approach is seeing whether the family is what needs amends, if there is friction or if there is a relationship in place. I see where they are in their relationship before moving forward*.” (Clergy#001) Another clergy described helping the family understand the patient’s legacy to find meaning to move forward:

*I help the family member also understand that once a story ends, there is a new story that is starting. It is a chapter, and new chapter is ready to start. What can you learn from them to be a better person and how can you let their legacy live on through you. -Clergy#001*

*Theme 2*: Caregivers reported spending time and being present as the primary way chaplains provided spiritual care. Providing prayer was common practice by both chaplains and clergy.

Caregivers were largely positive about their experiences with spiritual care in hospice, describing chaplain and clergy as caring and attentive. Caregivers reported that spending time and being present were typical ways that chaplains delivered spiritual care. A caregiver said, “*When the chaplain came, he would ask me how I was doing weekly–emotionally and spiritually, and if I needed anything. It wasn’t that it was from the Bible, but just checking on me*.”(Caregiver#0108) Another caregiver described the sensitive way in which the chaplain served their family:

*We are not Baptist, and our beliefs differ from the majority here. The chaplain was aware of our beliefs and came to support us in the kindliest manner. We did not get into a philosophical conversation. It would have annoyed us if he would have talked about heaven and that she was in a better place. That is not what we believe. He was in tune and intuitive to what we needed and wanted.–Caregiver#0201*

Caregivers expressed that prayer was an important part of spiritual care from both chaplains and clergy. One caregiver said,*“[The chaplain] prayed with us, which is what we needed. If we didn’t have a congregation, we would have had her over more and needed her more*.”(Caregiver#0202) Another caregiver described similar care from clergy, saying, *“[Clergy] prayed with her, holding her hands. It was intimate and appreciated making her feel here, present, that she was human and gave her dignity*”-(Caregiver#108).

Medical records provided another lens on the strategies utilized by chaplains and clergy. We found similar patterns of strategies in medical records for those who completed interviews and those who did not (Table 3). Medical records (as expected) identified few to no instances of making connections or active rapport building. Still, they documented chaplain and clergy being present and spending time with both patient and caregiver. About half of the records detailed provision of spiritual care resources (that is, handouts, references, etc.), and about a third showed evidence of attentive listening and recalling life experiences.

**Table 3:** Hospice team contributions to spiritual care, by QOL domain, overall (*n* = 23) and for caregivers interviewed or not, as documented in patient medical records

Spiritual care, by QOL domain and subdomains	Interviewed (*n* = 12)	Not interviewed (*n* = 11)	Overall (*n* = 23)
Hospice Environment	25% (3)	36% (4)	30% (7)
Calm peaceful environment	0% (0)	9% (1)	4% (1)
Music	25% (3)	18% (2)	22% (5)
Safe space	0% (0)	27% (3)	13% (3)
Patient Physical Well-being and Symptoms	25% (3)	36% (4)	30% (7)
Function ability	25% (3)	27% (3)	26% (6)
Strength	8% (1)	9% (1)	9% (2)
Patient Social Well-being	67% (8)	82% (9)	74% (17)
Family distress/support	33% (4)	18% (2)	26% (6)
Roles and relationships	58% (7)	82% (9)	70% (16)
Patient Emotional Well-being	83% (10)	82% (9)	83% (19)
General distress	8% (1)	18% (2)	13% (3)
Cognition/Attention	17% (2)	18% (2)	17% (4)
Happiness/peace or comfort	42% (5)	9% (1)	26% (6)
Emotional distress	42% (5)	36% (4)	39% (9)
Control	8% (1)	0% (0)	4% (1)
Anxiety	25% (3)	27% (3)	26% (6)
Depression	0% (0)	9% (1)	4% (1)
Fear	8% (1)	0% (0)	4% (1)
Pain distress	25% (3)	36% (4)	30% (7)
Patient Spiritual Well-being	83% (10)	73% (8)	78% (18)
Transcendence, higher power, God	67% (8)	18% (2)	43% (10)
Coping (illness, uncertainty, in hospice, dying)	67% (8)	45% (5)	57% (13)
Negative	0% (0)	9% (1)	4% (1)
Positive	67% (8)	36% (4)	52% (12)
Spiritual distress	8% (1)	0% (0)	4% (1)
Finding meaning, peace or comfort	50% (6)	45% (5)	48% (11)
Religiosity	58% (7)	45% (5)	52% (12)
Prayer (incl. rosary)	58% (7)	36% (4)	48% (11)
Other rituals (that is, scripture reading)	42% (5)	45% (5)	43% (10)
Sacraments	0% (0)	18% (2)	9% (2)
Caregiver Social Well-being	100% (12)	73% (8)	87% (20)
Caregiver time, practical tasks	33% (4)	9% (1)	22% (5)
Roles and relationship to patient	67% (8)	55% (6)	61% (14)
Emotional distress/support	42% (5)	36% (4)	39% (9)
Caregiver Emotional Well-being	58% (7)	73% (8)	65% (15)
Grief	33% (4)	36% (4)	35% (8)
Healthy	33% (4)	18% (2)	26% (6)
Unresolved struggling	8% (1)	9% (1)	9% (2)
Presence at hospice/Caregiving	42% (5)	45% (5)	43% (10)
Happiness/peace or comfort	25% (3)	9% (1)	17% (4)
Emotional distress/support	17% (2)	36% (4)	26% (6)
Control	8% (1)	9% (1)	9% (2)
Anxiety	8% (1)	18% (2)	13% (3)
Fear	0% (0)	9% (1)	4% (1)
Caregiver Spiritual Well-being	67% (8)	64% (7)	65% (15)
Transcendence, higher power, God	17% (2)	9% (1)	13% (3)
Coping	67% (8)	55% (6)	61% (14)
Negative	17% (2)	0% (0)	9% (2)
Positive	58% (7)	55% (6)	57% (13)
Finding meaning, peace or comfort	17% (2)	27% (3)	22% (5)
Religiosity	8% (1)	18% (2)	13% (3)
Prayer (incl. rosary)	8% (1)	18% (2)	13% (3)
Other rituals (that is, scripture reading)	0% (0)	9% (1)	4% (1)

*Theme 3:* Chaplain and clergy viewed their role as primary supporters of a patient’s social and spiritual well-being. Yet, chaplains had a more expansive view of the support they provided, including support for patient emotional well-being and for caregiver emotional and spiritual well-being.

Both chaplains and clergy viewed their role as providing social and spiritual support to those in hospice (Table 4). Both highlighted the importance of supporting patients in managing relationships at the end of life, emphasizing prayer and scripture. A chaplain noted their approach which is the validated firesetting questionnaire (Gannon et al., 2023) F.I.R.E that stands for ***F****amily, ****I****nterest, ****R****eligion, ****E****xperiences*:

*I talk through F.I.R.E. “F” is for family, and talking about family opens up the dialogue and talking about them. It is a warm approach. I look at questions about, “I”, their Interests. I see what they are interested in and pursue that to learn more about who they are. Then it is finding out about anything Religious, the “R”. I ask about their religious or spiritual background, if they were raised in something or attended something. If they had bad experiences or good experiences, do they believe in prayer, what do they believe in terms of a higher power or God. I like to talk about “E”, Experiences. The patients love to talk about major experiences in their lives, stories, trips, those types of things-(*Chaplain#3)

**Table 4:** Chaplain and clergy perspectives on the spiritual care they provide, by QOL domains

Spiritual care, by Quality of Life (QOL) domain and subdomains	Chaplain (*n* = 5)	Clergy (*n* = 3)
Hospice Environment	60% (3)	33% (1)
Calm peaceful environment	20% (1)	33% (1)
Safe Space	20% (1)	0% (0)
Music	40% (2)	33% (1)
Patient Physical Well-being & Symptoms	20% (1)	33% (1)
Functional ability	20% (1)	33% (1)
Patient Social Well-being	100% (5)	67% (2)
Roles and relationships	100% (5)	67% (2)
Family distress/support	40% (2)	0% (0)
Patient Emotional Well-being	100% (5)	33% (1)
Happiness/peace or comfort	40% (2)	33% (1)
Emotional distress	100% (5)	0% (0)
Control	20% (1)	33% (1)
Anxiety	20% (1)	0% (0)
Depression	20% (1)	0% (0)
Fear	40% (2)	0% (0)
Pain distress	20% (1)	0% (0)
Grief and bereavement	60% (3)	0% (0)
Patient Spiritual Well-being	100% (5)	100% (3)
Transcendence, higher power, God	60% (3)	0% (0)
Coping with illness, uncertainty, with being in hospice, with dying	80% (4)	100% (3)
Negative coping	20% (1)	0% (0)
Positive coping	60% (3)	100% (3)
Suffering	60% (3)	0% (0)
Spiritual distress	60% (3)	0% (0)
Finding meaning, peace or comfort or hope	60% (3)	0% (0)
Religiosity	100% (5)	100% (3)
Contact clergy	100% (5)	0% (0)
Community/congregation visitation	80% (4)	33% (1)
Prayer (incl. rosary)	80% (4)	100% (3)
Other rituals or practices (repeated actions) (that is, scripture reading)	80% (4)	67% (2)
Sacraments	80% (4)	0% (0)
Caregiver Social Well-being	40% (2)	33% (1)
Caregiver burden (time, practical tasks)	0% (0)	0% (0)
Roles and relationships to patient	40% (2)	33% (1)
Caregiver Emotional Well-being	60% (3)	33% (1)
Presence at hospice/Caregiving	0% (0)	33% (1)
Emotional distress	40% (2)	0% (0)
Grief and bereavement	40% (2)	0% (0)
Caregiver Spiritual Well-being	100% (5)	33% (1)
Transcendence, higher power, God	0% (0)	0% (0)
Coping with illness, uncertainty, with being in hospice, with dying	40% (2)	0% (0)
Negative coping	0% (0)	0% (0)
Positive coping	40% (2)	0% (0)
Suffering	0% (0)	0% (0)
Spiritual distress	33% (1)	0% (0)
Finding meaning, peace or comfort or hope	33% (1)	0% (0)
Religiosity	60% (3)	33% (1)
Contact clergy	20% (1)	NA
Community/congregation visitation	0% (0)	33% (1)
Prayer (incl. rosary)	0% (0)	33% (1)
Other rituals or practices (repeated actions) (that is, scripture reading)	40% (2)	0% (0)

A clergy discussed their approach of emphasizing prayer and connection:

*In hospice, not all people are Christian. You have different races, creeds, colors, religions. It is about building relationship. You can find most things in relationship. Once you have it, then they open up to you and you learn the best way to communicate. For Christians, it is a lot of devotion, prayer, hymns. If they are not Christian, you have to first see if they believe in God, and then change how you talk. If the patient is not responding, then it is ministering to the caregiver. -Clergy#003*

Compared with clergy, chaplains took a more expansive view of their role, including support for patient emotional well-being and support for the emotional and spiritual well-being of caregivers. Chaplains provided grief and bereavement support and helped manage emotional distress for patients and caregivers. A chaplain said, “*For families, I ask what they need, ask questions, use a kind open tone, offer open-ended help, and provide grief support through the same techniques of listening, questions and providing help with their loss*.” (Chaplain#3).

The same chaplain also relayed the importance of grief and bereavement support for the family, which are not reflected in the clergy’s self-reported areas of emphasis. The chaplain stated, “*Also part of my job is to support the family in their process and acceptance of the death, their grief and then also their bereavement once their loved one has passed*” (Chaplain#3).

*Theme 4*: Caregivers reported that together, the hospice care team, church, and family all shared responsibility for patient and caregiver spiritual well-being and for caregiver social well-being. They also shared that their church congregations and own families were primarily responsible for patient social well-being and caregiver emotional well-being and felt that the broader hospice care team was responsible for the hospice environment and patient physical well-being.

Caregivers of African-American hospice patients reported that other hospice staff and care team members (not chaplains) were primarily responsible for ensuring the hospice had a calm and peaceful environment. The larger hospice organization, nurses, or social workers were explicitly mentioned most often in providing this type of care (Table 5). Similarly, hospice staff and care team members (not chaplains) were primarily responsible for attending to patient functional abilities (e.g., help with walking).

**Table 5:** Who contributed to spiritual care, by QOL domains, overall and by individual, as reported by caregivers

Aspects of spiritual care By QOL domain and subdomains	Hospice team overall	Chaplain	RN or SW	General Hospice reference	Church overall	Clergy	Church member(s)	Family/Caregiver overall^#^	Patient themselves*
Hospice Environment	50% (6)	8% (1)	33% (4)	42% (5)	8% (1)	0% (0)	8% (1)	8% (1)	NA
Calm peaceful environment	50% (6)	8% (1)	25% (3)	42% (5)	8% (1)	0% (0)	8% (1)	8% (1)	NA
Safe space	8% (1)	0% (0)	8% (1)	0% (0)	0% (0)	0% (0)	0% (0)	8% (1)	NA
Patient Physical Well-being & Symptoms	42% (5)	0% (0)	33% (4)	8% (1)	0% (0)	0% (0)	0% (0)	0% (0)	NA
Functional ability	42% (5)	0% (0)	33% (4)	8% (1)	0% (0)	0% (0)	0% (0)	0% (0)	NA
Patient Social Well-being	17% (2)	17% (2)	0% (0)	0% (0)	58% (7)	25% (3)	42% (5)	58% (7)	17% (2)
Roles and relationships	17% (2)	17% (2)	0% (0)	0% (0)	42% (5)	17% (2)	33% (4)	58% (7)	17% (2)
Family distress/support	0% (0)	0% (0)	0% (0)	0% (0)	17% (2)	8% (1)	8% (1)	0% (0)	0% (0)
Patient Emotional Well-being	50% (6)	33% (4)	33% (4)	0% (0)	33% (4)	8% (1)	25% (3)	25% (3)	25% (3)
Happiness/peace or comfort	25% (3)	8% (1)	17% (2)	0% (0)	8% (1)	0% (0)	8% (1)	0% (0)	0% (0)
Emotional distress/support	42% (5)	25% (3)	33% (4)	0% (0)	25% (3)	8% (1)	17% (2)	25% (3)	25% (3)
Patient Spiritual Well-being	67% (8)	67% (8)	8% (1)	17% (2)	50% (6)	33% (4)	33% (4)	67% (8)	58% (7)
Transcendence, higher power, God	8% (1)	8% (1)	0% (0)	0% (0)	17% (2)	0% (0)	17% (2)	17% (2)	17% (2)
Positive coping+	17% (2)	8% (1)	8% (1)	0% (0)	25% (3)	8% (1)	17% (2)	25% (3)	33% (4)
Spiritual distress/support	17% (2)	17% (2)	0% (0)	0% (0)	25% (3)	8% (1)	17% (2)	25% (3)	17% (2)
Religiosity	50% (6)	50% (6)	0% (0)	17% (2)	50% (6)	33% (4)	33% (4)	58% (7)	42% (5)
Prayer (incl. rosary)	50% (6)	50% (6)	0% (0)	8% (1)	50% (6)	33% (4)	33% (4)	42% (5)	42% (5)
Community/congregation visitation	8% (1)	8% (1)	0% (0)	8% (1)	17% (2)	8% (1)	17% (2)	8% (1)	0% (0)
Other practices (scripture reading)	8% (1)	8% (1)	0% (0)	8% (1)	17% (2)	0% (0)	17% (2)	33% (4)	42% (5)
Caregiver Social Well-being	25% (3)	8% (1)	17% (2)	8% (1)	25% (3)	0% (0)	25% (3)	17% (2)	NA
Caregiver time, practical tasks	17% (2)	0% (0)	17% (2)	8% (1)	17% (2)	0% (0)	17% (2)	8% (1)	NA
Caregiver Emotional Well-being	67% (8)	42% (5)	33% (4)	33% (4)	33% (4)	17% (2)	33% (4)	42% (5)	8% (1)
Presence at hospice/Caregiving	17% (2)	17% (2)	8% (1)	0% (0)	0% (0)	0% (0)	0% (0)	8% (1)	NA
Happiness/peace or comfort	8% (1)	0% (0)	8% (1)	0% (0)	8% (1)	0% (0)	8% (1)	0% (0)	0% (0)
Emotional distress/support	33% (4)	17% (2)	25% (3)	17% (2)	8% (1)	0% (0)	8% (1)	8% (1)	8% (1)
Grief and bereavement	33% (4)	17% (2)	0% (0)	25% (3)	17% (2)	17% (2)	17% (2)	25% (3)	0% (0)
Caregiver Spiritual Well-being	42% (5)	42% (5)	8% (1)	0% (0)	33% (4)	17% (2)	25% (3)	67% (8)	8% (1)
Transcendence, higher power, God	0% (0)	0% (0)	0% (0)	0% (0)	8% (1)	0% (0)	8% (1)	25% (3)	0% (0)
Positive coping+	8% (1)	8% (1)	8% (1)	0% (0)	17% (2)	8% (1)	8% (1)	17% (2)	8% (1)
Spiritual distress/support	8% (1)	8% (1)	0% (0)	0% (0)	8% (1)	0% (0)	8% (1)	8% (1)	0% (0)
Religiosity	42% (5)	42% (5)	0% (0)	0% (0)	33% (4)	17% (2)	25% (3)	25% (3)	0% (0)
Prayer (incl. rosary)	42% (5)	42% (5)	0% (0)	0% (0)	33% (4)	17% (2)	25% (3)	17% (2)	0% (0)
Community/congregation visitation	17% (2)	17% (2)	0% (0)	0% (0)	17% (2)	8% (1)	17% (2)	8% (1)	0% (0)
Other practices (scripture reading)	8% (1)	8% (1)	0% (0)	0% (0)	0% (0)	0% (0)	0% (0)	0% (0)	0% (0)

QOL = quality of life. RN = registered nurse. SW = social worker. * indicates the caregiver reported the patient themselves supplied the aspect of spiritual care. The headings with “Overall” are not the sum of individual types who provided spiritual care, but the total number of unique caregivers who mentioned the specific people supplying spiritual care in that setting (that is, hospice, church, family). ^***#***^ denotes that “Family/Caregiver Overall” includes the caregivers themselves, other family and the patient. + indicates positive coping includes coping with illness, uncertainty, with being in hospice, with dying

Most caregivers reported receiving support for *patient social well-being*, primarily through roles and relationships (that is, being present for the patient). This role was primarily supported by the church family and caregiver. The church family and congregation also supported patients in dealing with family distress. The focus of *caregiver social well-being* support was primarily described as reducing caregiver burden (that is, support with practical tasks). Nurses or social workers and the church family shared social well-being support for the caregiver. Comparing the providers of social well-being for patients and caregivers, we heard that *patients* received the most support from the church and their family, whereas *caregivers* received less support overall and roughly equal support from the hospice care team, church, and their family.

Patient emotional well-being was most supported by the hospice care team, but also was supported through their churches and families. Nurses, social workers, and chaplains were those who primarily supported patient emotional distress and did this at similar levels as the family/caregiver. The hospice care team supported patient happiness, peace, or comfort most often among all who attended to those in hospice. Caregivers were provided the most support for their emotional well-being, primarily by the hospice at large including the care team. Hospice care team members equally shared responsibility for supporting caregiver’s emotional distress. Grief and bereavement support was provided the most through the family/caregivers followed by the chaplain, the hospice generally, then by clergy, and church family. Patients and caregivers received similar types and amounts of emotional supports, though caregivers received additional support through grief and bereavement support provided primarily by the hospice care team after their loved one’s passing. Not surprising, caregivers did not describe receiving support from the hospice patient.

For hospice patients, the most support was offered for their spiritual well-being. More than half of caregivers reported support for the patient’s spiritual well-being from all three main sources: hospice care team, family, and their church. Prayer was the need most often met and was primarily attended to by chaplains, followed by family/caregiver, patient themselves, and church family. The church and family also attended to transcendence, positive coping, and spiritual distress for the patient. The hospice care team provided the most spiritual support for caregivers through prayer from the chaplain. In contrast, the family and the church provided a wider range of spiritual supports (including prayer) for the caregiver.

Caregivers reported similar relative levels of support from the hospice care team, their church, and family as they reported for patients; though caregivers reported slightly less support overall from all sources. Caregivers reported that patients required and received more support for their need in recognizing a higher power (God), positive coping, and religious rituals and practices (that is, rites, sacraments, scripture reading) in hospice than they themselves did as caregivers. Patients required and received the most support through prayer and scripture reading.

Medical records also described the expansive view chaplains discussed toward spiritual care. We found similar patterns of endorsed strategies in patient records for those patient/caregivers who completed interviews and those that did not (Table 3). More than half of the records indicated that the hospice care team (through chaplains, nurses, or social workers) were involved in providing spiritual care. Three in four records noted support for patient and caregiver social well-being, patient emotional well-being, and patient spiritual well-being. About two-thirds also noted support for caregiver emotional and spiritual well-being. The most common support noted in the records was supporting patient’s relationships with others (70%), followed by supporting caregiver’s relationships with others (61%), caregiver coping (61%), and patient coping (57%). Similar to chaplain and clergy perspectives discussed during interviews, records documented more aspects of spiritual care support across the QOL domains and subdomains that were provided to support patient well-being than to support caregiver well-being.

## Discussion

We found that chaplains and clergy were the main source of spiritual well-being in hospice for African-American hospice patients and their caregivers, yet chaplains and clergy used some different strategies to provide spiritual care. Both chaplains and clergy used active listening techniques for building rapport and making connections. Chaplains also focused on cultural sensitivity and open-ended questions, whereas clergy emphasized reconciliation and life review. Chaplains typically are getting to know patients and caregivers for the first time, whereas clergy tend to have previous connections and trust with patients. This aligns with documented spiritual care requirements and guidelines which highlight the many activities associated with high-quality spiritual care (National Hospice and Palliative Care Organization, 2022a). These include chaplains supporting spiritual care, coordinating end-of-life care, and bereavement support (Jeuland et al., 2017; Williams et al., 2004) and clergy preparing individuals spiritually for death, managing relationships and resolutions, and administering religious rites and rituals according to the individual’s beliefs (Braun & Zir, 2010). Thus, our finding is not unexpected or novel based on the typical roles of chaplain and clergy (Braun & Zir, 2010; Williams et al., 2004), but our findings extend this information by delineating the specific strategies used by those providing spiritual care in hospice. This finding also underscores the importance of integrating chaplain and clergy support into hospice care routines and processes because what clergy and chaplains provide together is a more comprehensive care approach for patient and caregiver (Reese & Brown, 1997).

Caregivers of African-American hospice patients most commonly valued chaplain presence and sensitivity, with prayer being a central element of spiritual care from both chaplains and clergy. Yet, we also heard that chaplains provided broader support to both patients and caregivers, including emotional and grief support, whereas clergy focused more exclusively on spiritual well-being. This underscores the importance of African-American hospice patients and their caregivers engaging with hospice chaplains (not only clergy) to support their broader emotional and spiritual needs that are heightened at the end of life, while still pointing to the need for ensuring that patients and caregivers are enabled in hospice to connect with and gain the unique support offered through clergy at end of life (Quigley & McCleskey, 2021; Quigley et al., 2024).

Spiritual needs were not solely met by chaplains and clergy. Caregivers reported that their own spiritual needs were often met by both non-chaplain hospice care team members and family members, with spiritual care (again) mainly through prayer. However, not unexpectedly, caregivers also felt that the non-chaplain hospice care team provided less overall spiritual support for them compared to what was provided to the hospice patients. Specifically, caregivers reported that patients overall received more spiritual support (e.g., prayer, scripture, connection to a higher power) compared to caregivers, who reported receiving more emotional and practical support, particularly from non-chaplain hospice care team members. This highlights the important roles that nurses and social workers play in supporting the whole person in hospice (Reese & Brown, 1997). Nurses and social workers were most often responsible for the hospice environment and physical needs and well-being of the patient, but contributed to all aspects of well-being—spiritual, emotional, and social—of hospice patients and their caregivers. This supports the evidence that interdisciplinary teams are crucial to the holistic aim of supporting not only the well-being of the hospice patient, but also the well-being of caregivers (O'Callaghan et al., 2020).

Caregivers reported that the social and emotional support provided to them during their loved one’s time in hospice came primarily from family and the broader church family, with some social support received from chaplains and clergy. This highlights the important and larger role that family and church play in caregivers’ social and emotional well-being in hospice (Sloan et al., 2021). This finding highlights the social and emotional support needed in hospice that is provided outside of the hospice by family and the church as they support patients and caregivers during their time in hospice and during the bereavement period (Sloan et al., 2021).

Notably, information in medical records reflected similar patterns of what types of spiritual care were provided and by whom, and showed patient’s spiritual care was documented as part of their spiritual care assessment and care plan. Not unexpectedly, we found that caregiver support was less frequently mentioned in records, although they did contain documentation on relationship building and emotional coping for patients and caregivers. This finding highlights that medical record review can be a source of information on spiritual care provision and revealed the activities and presence of care team support.

From all our data sources (that is, interviews with clergy, chaplains, and caregivers and medical records), we found that the interdisciplinary care team, including chaplains, nurses, and social workers, was collectively covering and responsible for patients’ emotional, social, physical, and spiritual well-being, whereas family (including caregivers) and the church (including clergy) primarily supported spiritual and social needs for patients and caregivers with clergy focused on spiritual well-being and social support.

### Limitations

Our study has limitations. We used best methodological practices to mitigate biases found in qualitative analyses. Nevertheless, these findings are limited to one hospice’s experience of providing spiritual care to African-American hospice patients, their caregivers, and clergy who consented to the study. Our goal was to include a diverse set of experiences in a hospice that predominantly served an African-American community. The sample size was small, limiting the generalizability of our findings. We were unable to conduct observations; therefore, our findings rely solely on individuals’ reports of experiences in hospice. Still, our study design of gaining the perspectives of each patient’s caregiver, their clergy, and hospice chaplain, and their medical record enabled a comprehensive, in-depth picture of African-American hospice patient and caregiver experiences and highlighted the sources of data collection needed for understanding spiritual care and how it supports well-being. We could not include a larger sample of hospices or of caregivers or interviews of other interdisciplinary hospice team members (that is, nurses), nor could we include a comparison group of White hospice decedents; thus, a broader, multi-site evaluation is warranted.

### Conclusion

Spiritual care that supports the well-being of African-American hospice patients and their caregivers came through multiple sources inside and outside of the hospice. Chaplains and clergy were the main source of spiritual well-being in hospice, primarily through their ability to spend time and be present. Yet, spiritual needs were not solely met by chaplains and clergy. Caregiver’s own spiritual needs were often met also by nurses, social workers, and family. Nurses and social workers were most often responsible for the hospice environment and physical needs and well-being of the patient, but were reported to contribute to all aspects of the well-being of African-American hospice patients and their caregivers. Also, caregivers’ own social and emotional support came primarily from family members and the broader church, with some social support from chaplains and clergy.

Taken together, we heard that spiritual care that supports the whole person, that is, spiritual, social, emotional, and physical well-being, included all members of the interdisciplinary care team in the hospice plus family, clergy, and the church body outside the hospice. This brings to mind the well-referenced proverb that states, “It takes a village…”. Now, this proverb, which is an Igbo and Yoruba proverb that exists in many different African languages and cultures, typically states, “It takes a village to raise a child” (Reupert et al., 2022), but its main point emphasizes the value that African cultures place on family and community and reflects the essence of what we heard about the provision of spiritual care that supports all aspects of well-being for African-American hospice patients and their caregivers. Hospice professionals, chaplains, community clergy, and policy makers need to (re-)examine and ensure that in hospice there is an emphasis on the whole person in the approach to care, and also specifically to providing spiritual care. Our findings highlight the need to ensure the core principle that is the foundation of hospice care: whole person care. To advance equitable hospice care for all hospice patients, including African-American hospice patients and their caregivers, spiritual care provision needs to include those inside and outside of the hospice and aim to support the patient’s and caregiver’s spiritual, social, emotional, and physical well-being.

## Data Availability

The data from the study is unavailable due to confidentiality.
